# Uhlmann curvature in dissipative phase transitions

**DOI:** 10.1038/s41598-018-27362-9

**Published:** 2018-06-29

**Authors:** Angelo Carollo, Bernardo Spagnolo, Davide Valenti

**Affiliations:** 1 Department of Physics and Chemistry, Group of Interdisciplinary Theoretical Physics, University of Palermo, Viale delle Scienze, Ed. 18, I-90128 Palermo, Italy; 2 Radiophysics Department, Lobachevsky State University of Nizhni Novgorod, 23 Gagarin Avenue, Nizhni, Novgorod 603950 Russia; 3 Istituto Nazionale di Fisica Nucleare, Sezione di Catania, Via S. Sofia 64, I-90123 Catania, Italy; 4 Istituto di Biomedicina ed Immunologia Molecolare (IBIM) “Alberto Monroy”, CNR, Via Ugo La Malfa 153, I-90146 Palermo, Italy

## Abstract

A novel approach based on the Uhlmann curvature is introduced for the investigation of non-equilibrium steady-state quantum phase transitions (NESS-QPTs). Equilibrium phase transitions fall invariably into two markedly non-overlapping categories: classical phase transitions and quantum phase transitions. NESS-QPTs offer a unique arena where such a distinction fades off. We propose a method to reveal and quantitatively assess the quantum character of such critical phenomena. We apply this tool to a paradigmatic class of lattice fermion systems with local reservoirs, characterised by Gaussian non-equilibrium steady states. The relations between the behaviour of the Uhlmann curvature, the divergence of the correlation length, the character of the criticality and the dissipative gap are demonstrated. We argue that this tool can shade light upon the nature of non equilibrium steady state criticality in particular with regard to the role played by quantum vs classical fluctuations.

## Introduction

A challenging new paradigm has recently been put forward by the discovery of novel types of quantum phase transitions (QPTs)^1^ occurring in non-equilibrium steady states (NESSs)^2–10^. A comprehensive picture and characterisation of dissipative NESS-QPTs is lacking, partly due to their nature lying in a blurred domain, where features typical of zero temperature QPTs coexists with properties typical of thermal phase transitions. Such a coexistence between quantum and classical fluctuations are to some extent reminiscent of quantum to classical crossovers in equilibrium QPTs, with a major striking difference: the remarkably sharp character of a truly critical phenomenon. 

A natural approach to the investigation of such a novel scenario would be to adapt tools used in the equilibrium settings. In this letter, we propose the use of the geometric phase (GP)^11,12^, and in particular its mixed state generalisation, the Uhlmann GP^13^, to investigate NESS-QPT. GPs, and related geometrical tools, such as the Bures metrics^14–16^, have been successfully applied in the analysis of many equilibrium phase transitions^17–20^. The Bures metrics have been employed in thermal phase transitions^21^, and QPTs, both in symmetry-breaking^17–20,22^ as well as in topological phase transitions^23^. GPs are at the core of the characterisation of topological phase transitions^24^, and have been employed in the description and detection of QPTs, both theoretically^25–33^ and experimentally^34^. The use of GP in QPTs can be heuristically understood as follows: QPTs are determined by dramatic structural changes of the system state, resulting from small variations of control parameters. When approaching a criticality, two infinitesimally close states on the parameter manifold, become increasingly statistically distinguishable, i.e. their geometric-statistical distance grows. Abrupt changes in the distance are accompanied by singularities of the state space curvature, which in turn determine GP instabilities on states traversing loops in the neighbourhood of the criticality^25–31^.

Due to their mixed state nature, the NESSs require the use of a definition of GP in the density operators domain. Among all possible approaches^13,35–39^, the Uhlmann GP^13^ stands out for its deep-rooted relation to information geometry and metrology^40^, whose tools have been profitably employed in the investigation of QPT and NESS-QPT^18,41,42^. Uhlmann holonomy and GP have been applied to the characterisation of both topological and symmetry breaking equilibrium QPT^43–48^. Many proposals to measure the Uhlmann GP have been put forward^49,50^, and demonstrated experimentally^51,52^.

Motivated by this, we introduce the mean Uhlmann curvature (MUC) and investigate its role in the characterisation of dissipative NESS-QPTs. The MUC, defined as the Uhlmann GP per unit area of a density matrix evolving along an infinitesimal loop, has also a fundamental interpretation in multiparameter quantum metrology: it marks the measurement incompatibility between independent parameters arising from the quantum nature of the underlying physical system^53^. In this sense, the MUC is a measure of “quantumness” in the *multi-parameter* estimation problem, and its singular behaviour responds only to quantum fluctuations occurring across a phase transition.

We apply these ideas to the physically relevant setting of fermionic quadratic dissipative Lioviullian models, some of which show rich NESS features^2,3,41,42,54,55^.

## Results

### The mean Uhlmann curvature

The Uhlmann GP relies on the idea of amplitude of a mixed state. Given a density operator *ρ* acting on a Hilbert space ${\mathcal H}$ of dimension *n*, an amplitude is an operator *w* satisfying $\rho =w{w}^{\dagger }$. This definition leaves a *U*(*n*) gauge freedom in the choice of *w*, because *w*′ = *wU*, for any *U* ∈ *U*(*n*), generates the same *ρ*.

Let *ρ*_*λ*(*t*)_ be a family of density matrices, with $\gamma \,:\,=\{\lambda (t)\in  {\mathcal M} ,t\in [0,T]\}$ a smooth closed path in a parameter manifold ${\mathcal M}$, and *w*_*λ*(*t*)_ is a corresponding path of amplitudes. To lift the *U*(*n*) gauge freedom, Uhlmann introduced a parallel transport condition on *w*_*λ*(*t*)_^13^. For a closed trajectory *ρ*_*λ*(*t*)_, initial and final amplitudes are related by a unitary transformation *w*_*λ*(*T*)_ = *w*_*λ*(0)_*V*_*γ*_. If the Uhlmann parallel transport condition is fullfilled, *V*_*γ*_ is a *holonomy*, i.e. a non-Abelian generalisation of the Berry phase^13^, and reads ${V}_{\gamma }={\mathscr{P}}{e}^{i{\oint }_{\gamma }A}$, with ${\mathscr{P}}$ being the path ordering operator and $A={\sum }_{\mu }\,{A}_{\mu }d{\lambda }_{\mu }$ the Uhlmann connection one-form. The Uhlmann GP is defined as ${\phi }^{U}[\gamma ]\,:\,={\rm{\arg }}({w}_{\lambda (0)},{w}_{\lambda (T)})={\rm{\arg }}\,{\rm{Tr}}({w}_{\lambda (0)}^{\dagger }{w}_{\lambda (0)}{V}_{\gamma })$.

The Uhlmann connection *A* can be derived from the ansatz^56^ ${\partial }_{\mu }w=\frac{1}{2}{L}_{\mu }w-iw{A}_{\mu }$, where ${\partial }_{\mu }\,:\,=\partial /\partial {\lambda }_{\mu }$, and *L*_*μ*_’s are Hermitian operators known as symmetric logarithmic derivatives, implicitly defined as the operator solutions of ${\partial }_{\mu }\rho \,=\,:\frac{1}{2}({L}_{\mu }\rho +\rho {L}_{\mu })$. Unless otherwise stated, we will assume that *ρ* is full-rank. If *ρ* is singular, *L*_*μ*_ and *A*_*μ*_ are not unique. However, we will show that any quantity of interest to us can be extended by continuity to singular *ρ*’s^57^. It follows also that *A*_*μ*_ are Hermitian operators obeying the transformation rule of a non-abelian gauge potential, $A\to {U}^{\dagger }AU+i{U}^{\dagger }dU$ under *w* → *wU*, while *L*_*μ*_ are gauge invariant. The Uhlmann curvature, defined as *F*_*μν*_ = ∂_*μ*_*A*_*ν*_ − ∂_*ν*_*A*_*μ*_ − *i*[*A*_*μ*_, *A*_*ν*_], is equal to the Uhlmann holonomy per unit area associated to an infinitesimal loop in ${\mathcal M}$, i.e. ${F}_{\mu \nu }={\mathrm{lim}}_{\varepsilon \to 0}\,i\frac{1-{V}_{{\gamma }_{\mu ,\nu }}}{{\varepsilon }^{2}}$, where *γ*_*μν*_ is the infinitesimal parallelogram spanned by two independent directions ${\hat{e}}_{\mu }\varepsilon$ and ${\hat{e}}_{\nu }\varepsilon$ in ${\mathcal M}$. We focus on the Uhlmann GP per unit area for an infinitesimal loop, i.e.

$${{\mathscr{U}}}_{\mu \nu }\,:\,=\mathop{\mathrm{lim}}\limits_{\varepsilon \to 0}\frac{{\phi }^{U}[{\gamma }_{\mu \nu }]}{{\varepsilon }^{2}}={\rm{Tr}}({w}_{\lambda (0)}^{\dagger }{w}_{\lambda (0)}{F}_{\mu \nu }).$$

Notice that, while *F* is gauge covariant, i.e. it transforms as $F\to {U}^{\dagger }FU$ under *w* → *wU*, ${{\mathscr{U}}}_{\mu \nu }$ is a gauge invariant, i.e. it depends only on the infinitesimal path *ρ*(*t*). In the gauge in which ${w}_{0}=\sqrt{\rho (0)}$, ${{\mathscr{U}}}_{\mu \nu }={\rm{Tr}}(\rho {F}_{\mu \nu })$ acquires the meaning of a *mean Uhlmann curvature* (MUC).

It can be shown that (see Methods)

1 $${{\mathscr{U}}}_{\mu \nu }=\frac{i}{4}{\rm{Tr}}\rho [{L}_{\mu },{L}_{\nu }].$$

The above expression bears a striking resemblance with a pivotal quantity of quantum metrology, the quantum Fisher information matrix, defined as ${J}_{\mu \nu }=\frac{1}{2}{\rm{Tr}}\rho \{{L}_{\mu },{L}_{\nu }\}$. The quantum Fisher information matrix determines a figure of merit of the estimation precision of parameters labelling a quantum state, known as the quantum Cramér-Rao bound^58,59^. Given a set of locally unbiased estimators $\hat{\lambda }$ of the parameters $\lambda \in  {\mathcal M}$, the covariance matrix ${\rm{Cov}}{(\hat{\lambda })}_{\mu \nu }=\langle ({\hat{\lambda }}_{\mu }-{\lambda }_{\mu })\,({\hat{\lambda }}_{\nu }-{\lambda }_{\nu })\rangle$ is lower bounded (in a matrix sense) as follows

2 $${\rm{Cov}}(\hat{\lambda })\ge {J}^{-1}.$$

For single parameter estimation, the Cramér-Rao bound can always be saturated by the projective measurement on the eigenbasis of the symmetric logarithmic derivative. However, in a multi-parameter scenario this is not always the case, due to the non-commutativity of measurements associated to independent parameters. Within the framework of quantum local asymptotic normality^60–62^, one can prove that the multi-parameter quantum Cramér-Rao bound is attainable iff ${{\mathscr{U}}}_{\mu \nu }=0$ for all *λ*_*μ*_, *λ*_*ν*_^53^. In this sense, ${{\mathscr{U}}}_{\mu \nu }$ marks the *incompatibility* between *λ*_*μ*_ and *λ*_*ν*_, and such incompatibility arises from the inherent quantum nature of the underlying physical system. For a two-parameter model, the discrepancy between the *attainable* multi-parameter bound and the quantum Cramér-Rao bound can be estimated by the ratio $\mathrm{2|}{{\mathscr{U}}}_{\mu \nu }|/{\rm{Det}}J$, and the MUC is upper bounded by (see Methods)

3 $$|{{\mathscr{U}}}_{\mu \nu }|\le \sqrt{{\rm{Det}}J}/2.$$

When saturated, bound (3) marks the *condition of maximal incompatibility*, in which the quantum indeterminacy in the estimation problem reaches the order of Det(*J*)^−1/2^, the same of the quantum Cramér-Rao bound (2).

### Dissipative quadratic models

We now investigate the scaling law of the MUC, in dissipative Markovian models whose dynamics are generated by a master equation of Lindblad type^63^

4 $$\frac{d\rho }{dt}={\mathscr{L}}\rho =-\,i[{\mathscr{H}},\rho ]+\sum _{\alpha }\,(2{{\rm{\Lambda }}}_{\alpha }\rho {{\rm{\Lambda }}}_{\alpha }^{\dagger }-\{{{\rm{\Lambda }}}_{\alpha }^{\dagger }{{\rm{\Lambda }}}_{\alpha },\rho \}).$$

The Hamiltonian is assumed quadratic in the fermion operators, i.e. ${\mathcal H} \,:\,={{\boldsymbol{\omega }}}^{T}H{\boldsymbol{\omega }}$, where ${\boldsymbol{\omega }}\,:\,={({\omega }_{1}\ldots {\omega }_{2n})}^{T}$ is a vector of Majorana operators: ${\omega }_{2k-1}\,:\,={c}_{k}+{c}_{k}^{\dagger }$, ${\omega }_{2k}\,:\,=i({c}_{k}-{c}_{k}^{\dagger })$, with *k* = 1 … *n*, where *c*_*k*_ and ${c}_{k}^{\dagger }$ are annihilation and creation operators. *H* = −*H*^*T*^ is a 2*n* × 2*n* Hermitian matrix. ${{\rm{\Lambda }}}_{\alpha }={{\boldsymbol{l}}}_{\alpha }^{T}{\boldsymbol{\omega }}$ are bath operators with ${{\boldsymbol{l}}}_{\alpha }\,:\,={({l}_{1}^{\alpha },\ldots ,{l}_{2n}^{\alpha })}^{T}\in {{\mathbb{C}}}^{2n}$.

The Liouvillian ${\mathcal L}$ can be diagonalised exactly, and under certain conditions^64^, it admits a unique NESS *ρ*, which is Gaussian. A Gaussian state is completely specified by its correlation matrix ${{\rm{\Gamma }}}_{jk}\,:\,=1/2{\rm{Tr}}\rho [{\omega }_{j},{\omega }_{k}]$. Let $\lambda \in  {\mathcal M}$ be the set of parameters on which *H* and ***l***_*α*_’s depend. Due to uniqueness, ${\mathcal M}$ parametrises the admissible NESS *ρ*(*λ*). The correlation matrix of the NESS is the solution of the (continuous time) Lyapunov equation *X*Γ + Γ*X*^*T*^ = *Y*, with $X\,:\,=4[iH+{\bf{R}}{\bf{e}}(M)]={X}^{\ast }$, and $Y\,:\,=-\,i8{\bf{I}}{\bf{m}}(M)={Y}^{\dagger }=-\,{Y}^{T}$, where ${M}_{jk}\,:\,={\sum }_{\alpha }\,{l}_{j}^{\alpha }{({l}_{k}^{\alpha })}^{\ast }={({M}^{\dagger })}_{jk}$ is called bath matrix.

In Methods, we show that for a generic Gaussian Fermionic state the MUC can be expressed in a parameter-independent way, as

5 $${\mathscr{U}}=\frac{i}{4}{\rm{T}}{\rm{r}}({\rm{\Gamma }}K\wedge K),$$

where *K* is the operator solution of the (discrete time) Lyapunov equation *d*Γ = Γ*K*Γ − *K*, which can be formally solved by $K=-\,{({\bf{1}}-{{\rm{Ad}}}_{{\rm{\Gamma }}})}^{-1}(d{\rm{\Gamma }})$, where ${{\rm{Ad}}}_{{\rm{\Gamma }}}(X)\,:\,={\rm{\Gamma }}X\,{{\rm{\Gamma }}}^{\dagger }$ is the adjoint action.

According to^64^, the condition of NESS uniqueness is ${\rm{\Delta }}\,:\,=2\,{{\rm{\min }}}_{j}\,{\bf{R}}{\bf{e}}({x}_{j})\ge 0$, where *x*_*j*_ is an eigenvalue of *X*, and Δ is the Liouvillian spectral gap. When this condition is met, any state will eventually decay into the NESS in a time scale $\tau \simeq 1/{\rm{\Delta }}$. In the thermodynamical limit *n* → ∞ a vanishing gap Δ(*n*) → 0 may be accompained, though not-necessarily, by non-differentiable properties of the NESS^2,65^. For this reason, the scaling of Δ(*n*) has been used as an indication of NESS criticality^65–69^. NESS-QPT has been investigated through the scaling of the Bures metrics^19^, whose super-extensivity has been connected to a vanishing Δ^41^.

A similar relation between the super-extensivity of the MUC and Δ is implied by the inequality $\parallel {\mathscr{U}}{\parallel }_{\infty }\le \parallel J{\parallel }_{\infty }/2=\parallel g{\parallel }_{\infty }$ (see Methods), i.e. $\frac{|{{\mathscr{U}}}_{\mu \nu }|}{n}\le \frac{{P}_{{\rm{\Gamma }}}}{{{\rm{\Delta }}}^{2}}{(\parallel dY{\parallel }_{\infty }+2\parallel dX{\parallel }_{\infty })}^{2}$, where ||*B*||_∞_ indicates the largest singular value of a matrix *B*, ${P}_{{\rm{\Gamma }}}\,:\,=\parallel {(1+{\rm{\Gamma }}\otimes {\rm{\Gamma }})}^{-1}{\parallel }_{\infty }$ and *g* is the Bures metric tensor, which, except in pathological cases^57^, is equal to *g* = *J*/4. This bound shows that if ${P}_{{\rm{\Gamma }}}\simeq {\mathscr{O}}(1)$, a scaling of $|{\mathscr{U}}|\propto {n}^{\alpha +1}$ entails a dissipative gap that vanishes at least as Δ ∝ *n*^−*α*/2^, providing a relation between the dynamical properties of the NESS-QPT and the MUC.

However, as stated above, the scaling of MUC does indeed signal the presence of a NESS-QPT, but provide also a way of revealing the quantum character of the fluctuations that drive the criticality. On the one hand, the relation between MUC and quantum nature of the underlying physical system is apparent from the expression (1). The MUC arises from the commutator of two SLD, and, as such, its super-extensive properties cannot arise from classical fluctuations, as in equilibrium thermal phase transitions, but can only arise as a consequence of non-commutativity of close-by density matrices *ρ*(*λ*) and *ρ*(*λ* + *dλ*). In this sense, ${\mathscr{U}}$ is a signature of criticality associated to quantum fluctuations, as it cannot be sensitive to criticality induced by classical fluctuations, i.e. those associated only to changes in eigenvalues and not eigenstates of the density matrices.

Moreover, the comparison between the scaling laws of the MUC and quantum Fisher Information provides a means to estimate the quantum vs classical contributions to the fluctuations driving the criticality. This comparison is quantified by the ratio $Q\,:\,=|{\rm{Det}}2{\mathscr{U}}|/{\rm{Det}}J$, which according to the inequality (3) is upper bounded by *Q* ≤ 1, hence its scaling law is at most $Q\sim {n}^{0}$. When the above scaling law is saturated, the *condition of maximal incompatibility* of the associated quantum estimation problem is asymptotically satisfied. This implies that, in the thermodynamic limit *n* → ∞, the quantum character of the fluctuations driving the criticality cannot be neglected.

Let’s apply the above analysis to a specific model, the boundary-driven spin-1/2 XY chain^2^. In this model, an open chain of spin-1/2 particles interacts via the *XY*-Hamiltonian,

6 $${H}_{XY}=\sum _{j=1}^{n-1}\,(\frac{1+\delta }{2}{\sigma }_{j}^{x}{\sigma }_{j+1}^{x}+\frac{1-\delta }{2}{\sigma }_{j}^{y}{\sigma }_{j+1}^{y})+\sum _{j=1}^{n}\,\lambda {\sigma }_{j}^{z},$$

where the ${\sigma }_{j}^{x,y,z}$ are Pauli operators acting on the spin on the *j*-th site. At each boundary, the chain is in contact with two different reservoirs, described by Lindblad operators ${{\rm{\Lambda }}}_{L}^{\pm }=\sqrt{{\kappa }_{L}^{\pm }}({\sigma }_{j}^{x}\pm i{\sigma }_{j}^{y})/2$ and ${{\rm{\Lambda }}}_{R}^{\pm }=\sqrt{{\kappa }_{R}^{\pm }}({\sigma }_{j}^{x}\pm i{\sigma }_{j}^{y})/2$. A Jordan-Wigner transform converts the system into a quadratic fermionic dissipative model with Gaussian NESS^2,66^. The system experiences different phases as the anisotropy *δ* and magnetic field *h* are varied. For $h < {h}_{c}\,:\,=|1-{\delta }^{2}|$ the chain exibits long-range magnetic correlations (LRMC) and high sensitivity to external parameter variations. For *h* > *h*_*c*_ and along the lines *h* = 0 and *δ* = 0 the model shows short-range correlations, with correlation function ${C}_{jk}\,:\,=\langle {\sigma }_{j}^{z}{\sigma }_{k}^{z}\rangle -\langle {\sigma }_{j}^{z}\rangle \langle {\sigma }_{k}^{z}\rangle$ exponentially decaying: *C*_*jk*_ ∝ exp − |*j* − *k*|/*ξ*, with ${\xi }^{-1}\simeq 4\sqrt{2(h-{h}_{c})/{h}_{c}}$. In both long and short range phases, the dissipative gap closes as ${\rm{\Delta }}={\mathscr{O}}({n}^{-3})$ in the thermodynamical limit *n* → ∞. The critical line *h* = *h*_*c*_, is characterised by power-law decaying correlations *C*_*jk*_ ∝ |*j* − *k*|^−4^, and ${\rm{\Delta }}={\mathscr{O}}({n}^{-5})$. Therefore, the scaling law of Δ cannot distinguish long and short range phases, and can only detect the actual critical line *h* = *h*_*c*_. Likewise, Δ does not identify the transition from the LRMC phase to the *δ* = 0 and *h* = 0 lines.

**Figure 1:**
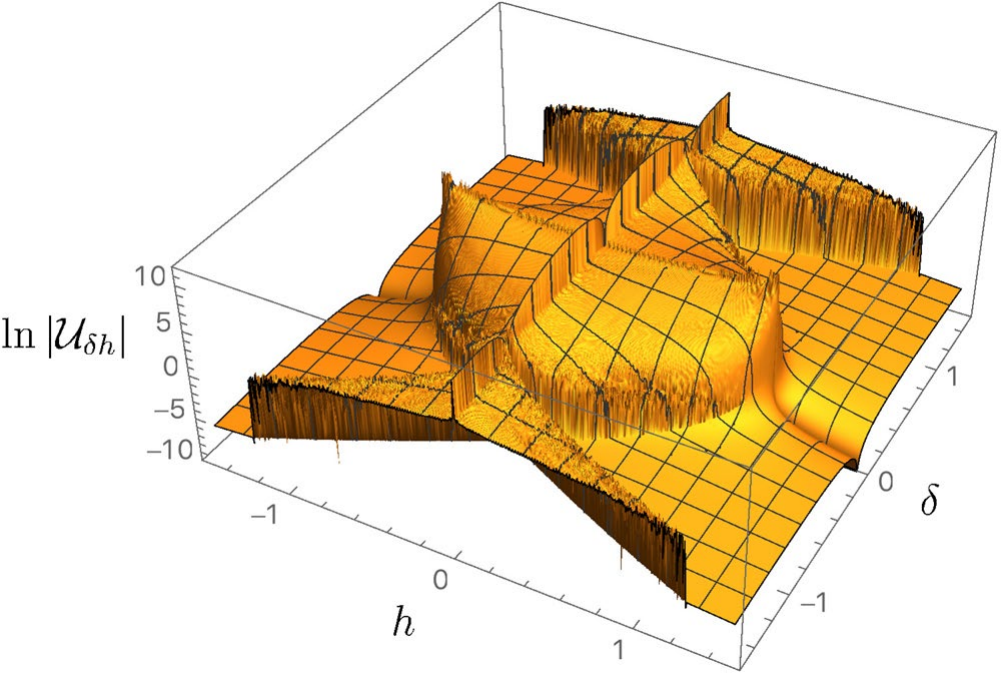
The MUC $|{{\mathscr{U}}}_{\delta h}|$ for the boudary driven XY model, for *n* = 300. The qualitative behaviour of MUC maps the phase diagram quite faithfully. The discontinuity accross the critical line $h=\,{h}_{c}\,:\,=|1-{\delta }^{2}|$ signals the transition between LRMC and short range phases. ${\kappa }_{L}^{+}=0.3$, ${\kappa }_{L}^{-}=0.5$,${\kappa }_{R}^{+}=0.1$, ${\kappa }_{R}^{-}=0.5$. The qualitative features remains unchanged for different values of ${\kappa }_{L,R}^{\pm }$.

**Figure 2:**
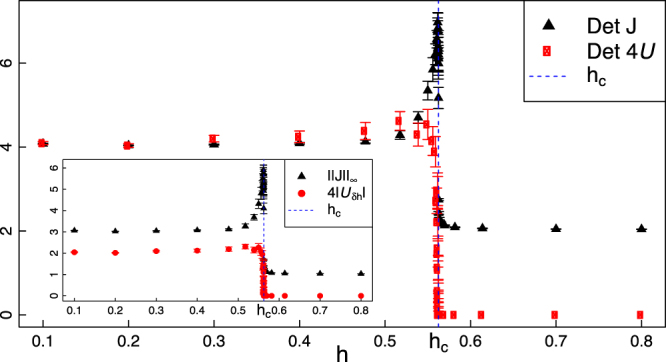
Boundary driven XY model. Scaling laws of the determinants (main) and maximal eigenvalues (inset) of the quantum Fisher information matrix *J* and mean Uhlmann curvature ${\mathscr{U}}$ for different values of *h*, with *δ* = 1.25 and *h*_*c*_ = |1 − *δ*^2^|. The laws do not depend on the particular values of the ${\kappa }_{R,L}^{\pm }$. The scalings are the results of fits on numerical data, with size ranging in *n* ∈ [20, 2000].

In Table 1, the MUC scaling law is compared with the scaling of ||*J*||_∞_, Det*J* and Δ in each region of the phase diagram. Figure 1 clearly shows that $|{{\mathscr{U}}}_{\delta h}|$ maps faithfully the phase diagram. A super-extensive behaviour of the MUC characterises the LRMC phase with a scaling $|{{\mathscr{U}}}_{\delta h}|={\mathscr{O}}({n}^{2})$, while in the short range phase the MUC is size independent. Thus, differently from Δ, the MUC discriminates these phases, with no need of crossing the critical line *h* = *h*_*c*_. Figure 2 shows that in the LRMC phase, the scaling law of the MUC saturates the upper bound (3), in contrast to the short range phase. This shows the striking different nature of the two phases. In the LRMC region, the system behaves as an inherently two-parameter quantum estimation model, where the parameter incompatibility cannot be neglected even in the thermodynamical limit. On the short-range phase, instead, the system is asymptotically quasi-classical. The critical line *δ* = 0 (with |*h*| ≤ *h*_*c*_) and the critical line *h* = 0, which mark regions of short range correlations embedded in a LRMC phase, show a MUC which grows super-extensively, with scaling ${\mathscr{O}}({n}^{3})$, and a nearly saturated inequality (3). In the critical line $h\simeq {h}_{c}$, despite the spectacular divergence of $\parallel J{\parallel }_{\infty }\simeq {\mathscr{O}}({n}^{6})$, the scaling law of $|{{\mathscr{U}}}_{\delta h}|$ drops to a constant, revealing an asymptotic quasi-classical behaviour of the model at the phase transition.

**Table 1:** Here we show a comparison between the scaling laws for: the dissipative gap Δ^2^, the largest eigenvalue ||*J*||_∞_ of the quantum Fisher information matrix^41^, the determinant of *J*, the largest eigenvalue $\parallel {\mathscr{U}}{\parallel }_{\infty }=|{{\mathscr{U}}}_{\delta h}|=\sqrt{{\rm{Det}}\,{\mathscr{U}}}$ of the MUC, and the ratio $Q\,:\,=|{\rm{Det}}2{\mathscr{U}}|/{\rm{Det}}J$ for each phase of the boundary driven XY model^2^.

Phase	Parameters	Δ	||*J*||_∞_	DetJ	|${{\mathscr{U}}}_{{\boldsymbol{\delta }}h}$|	Q
Critical	*h* = 0	*n* ^−3^	*n* ^6^	*n* ^7^	*n* ^3^	*n* ^−1^
Long range	0 < |*h*| < *h*_*c*_	*n* ^−3^	*n* ^3^	*n* ^4^	*n* ^2^	*n* ^0^
Critical	$h\simeq {h}_{c}$	*n* ^−5^	*n* ^6^	*n* ^7^	*n* ^0^	*n* ^−7^
Short range	*h* > *h*_*c*_	*n* ^−3^	*n*	*n* ^2^	*n* ^0^	*n* ^−2^
Critical	*δ* = 0, |*h*| < *h*_*c*_	*n* ^−3^	*n* ^2^	*n* ^8^	*n* ^3^	*n* ^−2^

The ratio *Q* ≤ 1 when *Q* ~ *n*^0^ marks the condition of maximal asymptotic incompatibility.

### Translationally invariant systems

An important subclass of quadratic Liouvillian Fermionic models are those enjoying the translational invariance symmetry. In such systems one can employ the whole wealth of powerful tools stemming out of the Fourier transform and work directly in the thermodynamical limit. This enables one to quantitatively define criticality in terms of singularities in the quasi-momentum space, thereby secluding the kinematics of the NESS-QPT from the dynamical properties of the model. The most natural notion of many-body criticality is in terms of diverging correlation length, which in a translationally invariant system is relatively straightforward to handle. This way of defining criticality enables one to bypass the difficulties arising from the ambiguous relation between NESS-QPTs and the vanishing dissipative gap.

The object of investigation is the covariance matrix, which in a translationally invariant system can be conveniently studied through its Fourier components. It is the non-analytical behaviour in the Fourier basis which conveys information on the long-wavelength limit, i.e. on the divergence of the correlation length.

Consider an explicit translationally invariant *d*-dimensional lattice of Fermions located at sites $r\in {{\mathbb{Z}}}_{L}^{d}$, and assume finite (or quasi-finite) range interaction. The system size is *n* = *L*^*d*^, and subsequently, one takes the thermodynamical limit *L* → ∞. One can define the covariance matrix over a discrete quasi-momentum space. However the considerations on the long-wavelength limit that will follow truly apply only at the thermodynamical limit: hence divergences of correlation lengths manifest genuine quantum many-body effects.

In a translationally invariant chain, the Fermions can be labelled as ***ω***_*r*_ = (*ω*_*r*,1_, *ω*_*r*,2_)^*T*^, where *ω*_*r*,*β*_ with *β* = 1, 2 are the two types of Majorana fermions on each site $r\in {\mathbb{Z}}$. The Hamiltonian can be written as ${\mathcal H} ={\sum }_{r,s}\,{{\boldsymbol{\omega }}}_{r}^{T}h(r-s){{\boldsymbol{\omega }}}_{s}$ and similarly the Lindbladians ${{\rm{\Lambda }}}_{\alpha }(r)={\sum }_{s}\,{{\boldsymbol{l}}}_{\alpha }^{T}(s-r){{\boldsymbol{\omega }}}_{s}$, where $h(r)=h{(r-)}^{\dagger }$ are 2 × 2 complex matrices and and ${{\boldsymbol{l}}}_{\alpha }(r)\in {{\mathbb{C}}}^{2}$. Accordingly, the bath matrix can be expressed as [*M*]_(*r*,*β*)(*s*,*β*′)_ = [*m*(*r* − *s*)]_*ββ*′_, (*β*, *β*′ = 1, 2), where $m(r)={m}^{\dagger }(\,-\,r)$ are the 2 × 2 matrices $m(r)\,:\,={\sum }_{\alpha ,s}\,{{\boldsymbol{l}}}_{\alpha }(s-r)\otimes {{\boldsymbol{l}}}_{\alpha }^{\dagger }(s)$.

In the limit of infinite large system, both Hamiltonian and bath matrix are circulant. And the correlation matrix of the unique steady state solution is circulant, too: ${[{\rm{\Gamma }}]}_{(r,\beta )(s,\beta ^{\prime} )}={[\gamma (r-s)]}_{\beta \beta ^{\prime} }:\,=1/2{\rm{Tr}}\rho [{\omega }_{r,\beta },{\omega }_{s,\beta ^{\prime} }]$. The Fourier component of the covariance matrix, called the covariance symbol, reads $\tilde{\gamma }(\varphi )\,:\,={\sum }_{r}\,\gamma (r){e}^{-i\varphi \cdot r}$, with *ϕ* ∈ [−*π*, *π*). In terms of the symbol functions, the continuous Lyapunov equation reduces to a set of 2 × 2 matrix equations

7 $$\tilde{x}(\varphi )\tilde{\gamma }(\varphi )+\tilde{\gamma }(\varphi ){\tilde{x}}^{T}(\,-\,\varphi )=\tilde{y}(\varphi ),$$

where $\tilde{x}(\varphi )=2[2i\tilde{h}(\varphi )+\tilde{m}(\varphi )+{\tilde{m}}^{T}(\,-\,\varphi )]$ and $\tilde{y}(\varphi )=-\,4[\tilde{m}(\varphi )-{\tilde{m}}^{T}(\,-\,\varphi )]$ are the symbol functions of *X* and *Y*, respectively, and $\tilde{h}(\varphi )$, $\tilde{m}(\varphi )={\sum }_{\alpha }\,{\tilde{{\boldsymbol{l}}}}_{\alpha }\otimes {\tilde{{\boldsymbol{l}}}}_{\alpha }^{\dagger }$ and ${\tilde{{\boldsymbol{l}}}}_{\alpha }(\varphi )$ are the Fourier components of *h*(*r*), *m*(*r*) and ***l***_*α*_(*r*), respectively. Notice that $\tilde{m}(\varphi )=\tilde{m}{(\varphi )}^{\dagger }={\sum }_{\alpha }\,{\tilde{{\boldsymbol{l}}}}_{\alpha }\otimes {\tilde{{\boldsymbol{l}}}}_{\alpha }^{\dagger }\ge 0$ is a positive semidefinite matrix. The spatial correlation between Majorana Fermions are then recovered from the inverse Fourier transform of the symbol function $\gamma (r)=\frac{1}{{(2\pi )}^{d}}\,{\int }_{{{\mathbb{T}}}^{d}}\,{d}^{d}\varphi \tilde{\gamma }(\varphi ){e}^{i\varphi \cdot r}$. Following^54,70^, here we will define criticality by the divergence of correlation length, which is defined as

8 $${\xi }^{-1}\,:\,=-\,\mathop{\mathrm{lim}}\limits_{|r|\to \infty }\frac{\mathrm{ln}\,\parallel \gamma (r)\parallel }{|r|}.$$

In the thermodynamical limt, the divergence may only arise as a consequence of the non-analytical dependence of *γ*(*r*) on the system parameters. Let’s confine ourselves to the case of a one-dimensional Fermionic chain. In order to derive informations on the large distance behaviour of the correlations, it is convenient to express the integral of the inverse Fourier transform in the complex plane, though the analytical continuation *e*^*iϕ*^ → *z*. This results in the following expression for the correlation function

9 $$\gamma (r)=\sum _{\bar{z}\in {S}_{1}}\,{{\rm{Res}}}_{\bar{z}}[{z}^{r-1}\tilde{\gamma }(z)],$$

where ${{\rm{Res}}}_{\bar{z}}$ indicates the residues of the poles inside the unit circle ${S}_{1}\,:\,=\{z||z|\le 1\}$. Since $\tilde{\gamma }(z)$ is the solution of a finite dimensional matrix equation (7), it may only possess simple poles. Thus, the above expression may become singular only when an isolated pole of $\tilde{\gamma }(z)$ approaches the unit circle from the inside^54,70^. This may happen for some specific critical values $\lambda ={\lambda }_{0}\in  {\mathcal M}$. As *λ* approaches *λ*_0_ the correlation length *ξ* diverges. One can show that the long wave-length behaviour is governed by the closest pole to unit circle $|{\bar{z}}_{0}|$, and indeed the correlation length is given by $\xi =\,\mathrm{ln}\,|{\bar{z}}_{0}|$.

### Mean Uhlmann Curvature and Criticality in Translationally Invariant Models

We will show that the MUC is sensitive to the criticality, but only in the sense of a truly diverging correlation length. Indeed one can show that the Uhlmann curvature is insensitive to the vanishing of the dissipative gap, if the latter, as it may happen, is not accompanied by a diverging correlation length. In this sense, the Uhlmann curvature confirms its role as a witness of the purely kinematic aspects of the criticality, and it is only indirectly affected by the dynamical features of the NESS-QPT.

Thanks to the translational symmetry, one can exploit the formalism of Fourier transform and derive a quite compact expression of the MUC. By applying the convolution theorem on the equation (5), one obtains the following expression for the MUC *per site*

10 $${\bar{{\mathscr{U}}}}_{\mu \nu }\,:\,=\mathop{\mathrm{lim}}\limits_{n\to \infty }\frac{{{\mathscr{U}}}_{\mu \nu }}{n}=\frac{1}{(2\pi )}\,{\int }_{-\pi }^{\pi }\,d\varphi \,{u}_{\mu \nu }(\varphi ),$$

where

11 $${u}_{\mu \nu }(\varphi )\,:\,=\frac{i}{4}{\rm{Tr}}\{\tilde{\gamma }(\varphi )[{\kappa }_{\mu }(\varphi ),{\kappa }_{\nu }(\varphi )]\}=\frac{i}{4}{\rm{Tr}}\{{\kappa }_{\nu }(\varphi )[\tilde{\gamma }(\varphi ),{\kappa }_{\mu }(\varphi )]\},$$

In the above expression, *κ*_*μ*_(*ϕ*) is the operator solution of the 2 × 2 discrete time Lyapunov equation

12 $${\partial }_{\mu }\tilde{\gamma }(\varphi )=\tilde{\gamma }(\varphi ){\kappa }_{\mu }(\varphi )\tilde{\gamma }(\varphi )-{\kappa }_{\mu }(\varphi ).$$

In the eigenbasis of $\tilde{\gamma }(\varphi )$, with eigenvalues ${\tilde{\gamma }}_{j}$, the explicit solution of (12) reads ${({\kappa }_{\mu }(\varphi ))}_{jk}=\frac{{({\partial }_{\mu }\tilde{\gamma }(\varphi ))}_{jk}}{1-{\tilde{\gamma }}_{j}{\tilde{\gamma }}_{k}}$. Notice that the diagonal terms (*κ*_*μ*_(*ϕ*))_*jj*_ provide vanishing contributions to eq. (11) (they commute with $\tilde{\gamma }(\varphi )$). Hence, eq. (11) can be cast in the following basis independent form

13 $${u}_{\mu \nu }(\varphi )=\{\begin{array}{cc}\frac{i}{4}\frac{{\rm{T}}{\rm{r}}\{\mathop{\gamma }\limits^{ \sim }(\varphi )[{{\rm{\partial }}}_{\mu }\mathop{\gamma }\limits^{ \sim }(\varphi ),{{\rm{\partial }}}_{\nu }\mathop{\gamma }\limits^{ \sim }(\varphi )]\}}{{[1-{\rm{D}}{\rm{e}}{\rm{t}}\mathop{\gamma }\limits^{ \sim }(\varphi )]}^{2}} & {\rm{D}}{\rm{e}}{\rm{t}}\mathop{\gamma }\limits^{ \sim }(\varphi )\ne 1\\ 0 & {\rm{D}}{\rm{e}}{\rm{t}}\mathop{\gamma }\limits^{ \sim }(\varphi )=1\end{array}.$$

Notice that the condition ${\rm{Det}}\tilde{\gamma }(\varphi )=1$ is equivalent to having two eigenvalues of correlation matrix equal to (*γ*_*i*_, *γ*_*k*_) = ±(1, 1). Such extremal values cause no singularity in MUC, but result in a vanishing contribution to the MUC.

In Methods, we will demonstrate that a singularity of $\bar{{\mathscr{U}}}$ signals the occurrence of a criticality. Specifically, employing the analytical extension in the complex plane of *u*_*μν*_(*ϕ*) leads to

14 $${\bar{{\mathscr{U}}}}_{\mu \nu }=\sum _{\bar{z}^{\prime} \in {S}_{1}}\,{{\rm{Res}}}_{\bar{z}^{\prime} }[{z}^{-1}{u}_{\mu \nu }(z)].$$

Notice that *u*_*μν*_(*z*) has at most isolated poles, due to its rational dependence on *z*. Assume that as $\lambda \to {\lambda }_{0}\in  {\mathcal M}$, a pole ${\bar{z}}_{0}$ of *u*_*μν*_(*z*) approaches the unit circle from inside, which is the only condition under which $\bar{{\mathscr{U}}}$ is singular in *λ*_0_. One can show that, whenever a pole ${\bar{z}}_{0}$ of *u*_*μν*_(*z*) approaches the unit circle, also a pole $\bar{z}$ of $\tilde{\gamma }(z)$ approaches the same value, causing the correlation length to diverge. Therefore the singular behaviour of the Uhlmann phase necessarily represents a sufficient criterion for a NESS-QPT. Notice also (see Methods) that such criticalities are necessarily accompanied by the closure of the dissipative gap, however, the converse is in general not true. Indeed, *a vanishing dissipative gap is not a sufficient condition for criticality*, but only necessary. This fact can be readily checked with the model discussed in the next subsection, which shows a closing dissipative gap without the occurrence of a diverging correlation length.

**Figure 3:**
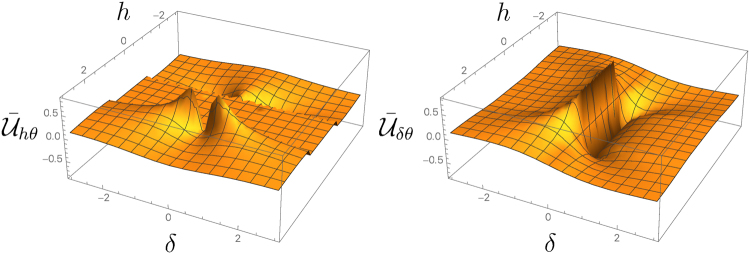
The mean Uhlmann curvature per number of sites $\bar{{\mathscr{U}}}$ for the rotated XY model with local reservoirs. The dependence of ${\bar{{\mathscr{U}}}}_{h\theta }$ (left) and of ${\bar{{\mathscr{U}}}}_{\delta \theta }$ (right) on the parameters *δ* and *h*. The mean Uhlmann curvature shows a singular behaviour in the critical regions of the model. ${\bar{{\mathscr{U}}}}_{h\theta }$ is discontinuous in the *XY* critical points |*h*| = 1, and ${\bar{{\mathscr{U}}}}_{\delta \theta }$ is discontinuous in the *XX* type criticalities *δ* = 0, |*h*| < 1.

Moreover, a singularity in the MUC may only arise as the result of criticality and are otherwise insensitive to a vanishing dissipative gaps. These features are exemplified in the following translational invariant dissipative fermionic chain: the rotated XY model with periodic boundary conditions^25,26^, $H=R(\theta ){H}_{XY}R{(\theta )}^{\dagger }$, with $R(\theta )={e}^{-i\frac{\theta }{2}{\sum }_{j}{\sigma }_{j}^{z}}$ and

15 $${H}_{XY}=\sum _{j=1}^{n}\,(\frac{1+\delta }{2}{\sigma }_{j}^{x}{\sigma }_{j+1}^{x}+\frac{1-\delta }{2}{\sigma }_{j}^{y}{\sigma }_{j+1}^{y}+\lambda {\sigma }_{j}^{z}),$$

where each site *j* is coupled to two local reservoirs with Lindblad operators ${{\rm{\Lambda }}}_{j}^{\pm }=\varepsilon \mu {\sigma }_{j}^{\pm }$. The spin-system is converted into a quadratic fermionic model via Jordan-Wigner transformations. The Liouvillian spectrum can be solved exactly^2,64,68^ and it is independent of *θ*. In the weak coupling limit *ε* → 0, the symbol function of the NESS correlation matrix reads $\tilde{\gamma }(\varphi )={{\boldsymbol{\gamma }}}^{T}\cdot {\boldsymbol{\sigma }}$, where ${\boldsymbol{\sigma }}\,:\,={({\sigma }_{x},{\sigma }_{y},{\sigma }_{z})}^{T}$, and ${\boldsymbol{\gamma }}=g{[t(\varphi )\cos \theta ,-1,t(\varphi )\sin \theta ]}^{T}$, with $g=\frac{{\nu }^{2}-{\mu }^{2}}{{\nu }^{2}+{\mu }^{2}}\frac{1}{1+t{(\varphi )}^{2}}$ and $t(\varphi )\,:\,=\delta \,\sin \,\varphi /(\cos \,\varphi -h)$. The system shows criticality in the same critical regions of the *XY* hamiltonian model^68^. By using expression (14) we can calculate the exact values of the mean Uhlmann curvature. We find that ${\bar{{\mathscr{U}}}}_{\delta h}$ vanishes, while ${\bar{{\mathscr{U}}}}_{\delta \theta }$ and ${\bar{{\mathscr{U}}}}_{h\theta }$ are plotted in Fig. 3. As predicted, the Uhlmann curvature shows a singular behaviour only across criticality. In particular, ${\bar{{\mathscr{U}}}}_{h\theta }$ is discontinuous in the *XY* critical points |*h*| = 1, while ${\bar{{\mathscr{U}}}}_{\delta \theta }$ is discontinuous in the *XX* type criticalities *δ* = 0, *h* < 1.

### A model with closing dissipative gap without criticality

**Figure 4:**
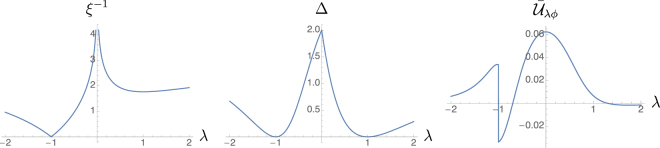
Model of a 1D fermionic chain on a ring showing a closing dissipative gap that does not imply a diverging correlation length. This is the model discussed in the last subsection of Results, which is a simple extension of a model introduced in^71^. The inverse correlation length, the dissipative gap and the MUC are shown, respectevely, from the left to the right panel. The model is critical only for *λ* = −1, while the gap closes for both *λ* = ±1. As expected, the discontinuity of MUC captures the criticality, and it is otherwise insensitive to a vanishing gap.

In this section we will show an example of a 1D fermionic dissipative system in which the closure of the dissipative gap does not necessarily lead to a diverging correlation length. Consider a chain of fermions on a ring geometry, with *no Hamiltonian* and a reservoir defined by the following set of Lindblad operators

$${\rm{\Lambda }}(r)=[(1+\lambda ){{\boldsymbol{l}}}_{0}^{T}{{\boldsymbol{\omega }}}_{r}+{{\boldsymbol{l}}}_{1}^{T}{{\boldsymbol{\omega }}}_{r+1}+\lambda {{\boldsymbol{l}}}_{2}^{T}{{\boldsymbol{\omega }}}_{r+2}]/n(\lambda ),$$

where *r* = 1, …, *n*, ***l***_0_ = (cos *θ*, −sin *θ*)^*T*^, ***l***_1_ = ***l***_2_ = *i*(sin *θ*, cos *θ*)^*T*^, and *n*(*λ*) = 4(*λ*^2^ + *λ* + 1), with $\lambda \in {\mathbb{R}}$, *θ* = [0, 2*π*). This is a simple extension of a model introduced in^71^, which, under open boundary conditions, shows a dissipative topological phase transition for *λ* = ±1. In the thermodynamical limit *n* → ∞, the eigenvalues of $\tilde{x}(\varphi )$ are *x*_1_ = 4(1 + *λ*)^2^/*n*(*λ*)^2^, and *x*_2_ = 4(1 + 2*λ* cos *ϕ* + *λ*^2^)/*n*(*λ*)^2^, showing a closure of the dissipative gap at *λ* = ±1. For |*λ*| ≠ 1 the unique NESS is found by solving the continuous Lyapunov equation (7). The symbol function, in a Pauli matrix representation, results $\tilde{\gamma }(\varphi )={\boldsymbol{\gamma }}\cdot {\boldsymbol{\sigma }}$, with

$${\boldsymbol{\gamma }}=g(\varphi )[\begin{array}{c}(\sin \,\varphi +\lambda \,\sin \,2\varphi )\,\cos \,2\theta \\ (\cos \,\varphi +\lambda \,\cos \,2\varphi )\\ -(\sin \,\varphi +\lambda \,\sin \,2\varphi )\,\sin \,2\theta \end{array}],$$

where *g*(*ϕ*) = (1 + *λ*)/(1 + *λ* + *λ* cos *ϕ* + *λ*^2^), with eigenvalues ±$g(\varphi )\sqrt{1+{\lambda }^{2}+2\lambda \,\cos \,\varphi }$. This shows that $\tilde{\gamma }$ is critical in the sense of diverging correlation, only for *λ* = −1 and not for *λ* = 1, even if the dissipative gap closes in both cases. Figure 4 shows the dependence of the inverse correlation length of the bulk, the dissipative gap and the mean Uhlmann curvature ${\bar{{\mathscr{U}}}}_{\lambda \varphi }$ on the parameter *λ*. Notice a discontinuity of the Uhlmann phase corresponding to the critical point *λ*_0_ = −1, while it does not show any singularity for *λ* = 1 where the gap closes.

## Discussion

We have introduced an entirely novel approach to quantitatively assess the “quantum-ness” of critical phenomena. To this end, we resorted to ideas borrowed from quantum estimation theory, which endow the geometric phase approach with an operationally well defined character. The geometrical interpretation offers an intuitive explanation as to why singularities of MUC emerge in criticalities, and leads to a unified interpretation for equilibrium and out-of-equilibrium QPTs. In quantum metrology, the MUC accounts for the discrepancy between an inherently quantum and a quasi-classical multi-parameter estimation problem, shading a new light onto the nature of correlations in NESS-QPTs. We have explored the properties of the MUC in the physically relevant class of dissipative NESS-QPTs exhibited by quadratic fermionic Liouvillian models. A relation between the singular behaviour of the MUC and the criticality has been analytically demonstrated. We have employed specific prototypical models, showing that the scaling laws and the singularities of ${\mathscr{U}}$ map faithfully the phase diagrams. This approach goes well beyond the application to the important class of quadratic dissipative models analysed here, and introduces a tool suitable for the systematic investigation of out-of-equilibrium quantum critical phenomena. It immediately extends to phase transitions with and without order parameters, quenched dynamics in open and closed systems, topological dissipative phase transitions, dynamical critical phenomena. Moreover, this idea is also a promising tool which may glean insight on the interplay between competing orders both in equilibrium and non-equilibrium QPTs.

## Methods

### Uhlmann geometric phase and mean Uhlmann curvature

Here we will briefly review the idea of the Uhlmann geometric phase, and derive the expression of the mean Uhlmann curvature as a function of the symmetric logarithmic derivatives (SLDs). Given a density operator *ρ* acting on a Hilbert space ${\mathcal H}$ of dimension *n*, an exteded Hilbert space is defined by attaching an ancilla *a*: ${ {\mathcal H} }_{{\rm{ext}}}= {\mathcal H} \otimes { {\mathcal H} }^{a}$. A purification is defined as any pure state $\psi \in  {\mathcal H}$ such that *ρ* = Tr_*a*_|*ψ*〉〈*ψ*|, where Tr_*a*_ is the partial trace over the ancilla. A standard choice for ${ {\mathcal H} }^{a}$ is the dual of ${\mathcal H}$, then ${ {\mathcal H} }_{{\rm{ext}}}$ becomes the space of operator *w* over ${\mathcal H}$, with Hilbert-Schmidt scalar product $(w,v)\,:\,={\rm{Tr}}({w}^{\dagger }v)$. Hence, a purification can be equivalently expressed in terms of any Hilbert-Schmidt operators *w*, called *amplitudes*, such that

16 $$\rho =w{w}^{\dagger }$$

The above equation, leaves a gauge freedom *U*(*n*) in the choice of *w*, as any *w*′ = *wU* is an amplitude of the same *ρ*. Indeed, from the polar decomposition theorem we can always uniquely parametrise an amplitude as $w=\sqrt{\rho }U$.

Given a pure state *ψ*, a similar *U*(1) gauge freedom is obtained by the simple observation that any *ψ* = *e*^*iφ*^*ψ*′ represents the same element of the projective Hilbert space. Let |*ψ*_*λ*_〉〈*ψ*_*λ*_| be a family of pure states parameterised by $\lambda \in  {\mathcal M}$, and let $\gamma \,:\,=\{\lambda (t)\in  {\mathcal M} ,t\in [0,T]\}$ be a smooth closed path in the parameter manyfold ${\mathcal M}$. Given such a family we can choose any representative trajectory ${\psi }_{\lambda (t)}={e}^{i\phi (t)}{\psi }_{\lambda (t)}^{^{\prime} }$ in the Hilbert space. If the trajectory chosen fulfils the prescription of parallel transport, i.e. $\langle {\psi }_{\lambda (t)}|\frac{d}{dt}|{\psi }_{\lambda (t)}\rangle =0$, then the phase difference *φ*^*B*^ between initial and final state $|{\psi }_{\lambda (T)}\rangle ={e}^{i{\phi }^{B}}|{\psi }_{\lambda (0)}\rangle$ is purely geometric in nature, i.e. it solely depends on the path *γ*, regardless of parameterisation and re-gauging. This phase is called Berry phase and its value reads ${\phi }^{B}={\oint }_{\gamma }\,{A}^{B}$, where ${A}^{B}\,:\,={\sum }_{\mu }\,{A}_{\mu }^{B}d{\lambda }_{\mu }$ is the Berry connection one-form, whose components are ${A}_{\mu }^{B}\,:\,=i\langle {\psi }_{\lambda }|{\partial }_{\mu }|{\psi }_{\lambda }\rangle$, where ${\partial }_{\mu }\,:\,=\partial /\partial {\lambda }_{\mu }$. By exploiting the Stokes theorem, we can convert the loop integral of *A*^*B*^ to an integral ${\phi }^{B}={\int }_{S}\,{F}^{B}$ over a surface *S* bounded by the path *γ*, where ${F}^{B}\,:\,=d{A}^{B}=\frac{1}{2}\,{\sum }_{\mu \nu }\,{F}_{\mu \nu }^{B}d{\lambda }_{\mu }\wedge d{\lambda }_{\nu }$ is the Berry curvature two-form, whose components are ${F}_{\mu \nu }^{B}\,:\,={\partial }_{\mu }{A}_{\nu }^{B}-{\partial }_{\nu }{A}_{\mu }^{B}$. The parallel transport condition is equivalent to choose the representative path *ψ*_*λ*(*t*)_ that minimizes the length of the path on the Hilbert space measure by $l={\int }_{0}^{T}\,d\tau \sqrt{\langle \dot{\psi }(\tau )|\dot{\psi }(\tau )\rangle }$.

Similarly, we can have a smooth closed trajectory of density matrices, *ρ*_*λ*(*t*)_, parametrised by a path *γ*: $\lambda (t)\in  {\mathcal M}$, *t* ∈ [0, *T*], and, correspondingly, a path of Hilbert-Schmidt operators *w*_*λ*(*t*)_ in ${ {\mathcal H} }_{{\rm{ext}}}$. The choice of amplitudes is quite redundant due to the local *U*(*n*) gauge freedom. Similarly to the pure state case, this redundancy can be mitigated by imposing the so called Uhlmann parallel transport condition, which prescribes that, given any two *ρ*_1_ and *ρ*_2_, their respective amplitudes *w*_1_ and *w*_2_ are parallel whenever

17 $${w}_{1}^{\dagger }{w}_{2}={w}_{2}^{\dagger }{w}_{1}\ge 0.$$

This equivalently means that the chosen *w*_1_ and *w*_2_ are those that maximise their Hilbert Schmidt scalar product $({w}_{1},{w}_{2})\,:\,={\rm{Tr}}({w}_{1}^{\dagger }{w}_{2})$, i.e.

$$({w}_{1},{w}_{2})=\mathop{max}\limits_{{{w}^{{\rm{^{\prime} }}}}_{2}}\,|({w}_{1},{{w}^{{\rm{^{\prime} }}}}_{2})|\,:\,={\mathscr{F}}({\rho }_{1},{\rho }_{2})$$

where the maximum is taken over all ${w^{\prime} }_{2}$ purifying *ρ*_2_. The above maximal value depends on *ρ*_1_ and *ρ*_2_ only, and it is equal to ${\mathcal F} ({\rho }_{1},{\rho }_{2})={\rm{Tr}}\sqrt{\sqrt{{\rho }_{1}}{\rho }_{2}\sqrt{{\rho }_{1}}}$, the so called Uhlmann fidelity of *ρ*_1_ and *ρ*_2_. Through the fidelity one can define a geometric measure of statistical indistinguishability between states *ρ*_1_ and *ρ*_2_^16^, the Bures distance

$${d}_{B}^{2}({\rho }_{1},{\rho }_{2})\,:\,=2[1-{\mathscr{F}}({\rho }_{1},{\rho }_{2})].$$

which, for infinitesimally closed states, defines a Riemannian metrics on the manifold of density operators, the Bures metrics

$$\sum _{\mu \nu }\,{g}^{\mu \nu }d{\lambda }_{\mu }d{\lambda }_{\mu }\,:\,={d}_{B}^{2}({\rho }_{\lambda },{\rho }_{\lambda +d\lambda }).$$

Applied to any two neighbouring points *w*_*λ*(*t*)_ and *w*_*λ*(*t*+*dt*)_ of a smooth path of amplitudes, the parallel transport condition (17) becomes

18 $${w}^{\dagger }\dot{w}-{\dot{w}}^{\dagger }w=0,$$

where dots denote derivatives with respect to *t*. The maximisation of the overlap (*w*_*λ*(*t*)_, *w*_*λ*(*t*+*dt*)_) is equivalent to the minimisation of the “velocity” $v\,:\,=\sqrt{(\dot{w},\dot{w})}$, which in turns means that the path of amplitudes fullfilling the Uhlmann condition are those with the shortest length, measured by $l\,:\,={\int }_{0}^{T}\,d\tau \sqrt{({\dot{w}}_{\lambda (\tau )},{\dot{w}}_{\lambda (\tau )})}$.

According to^56^, the parallel transport condition (18) is fullfilled by the following ansatz

19 $$\dot{w}=\frac{1}{2}{L}_{t}w,\,{L}_{t}^{\dagger }={L}_{t}.$$

*L*_*t*_ can be determined by differentiating $\rho =w{w}^{\dagger }$ and inserting (19), which yields

20 $$\dot{\rho }=\frac{1}{2}\{{L}_{t},\rho \},$$

where {., .} is the anticommutator. *L*_*t*_, known as the symmetric logarithmic derivative (SLD), is implicitly defined as the (unique) operator solution of (20) with the auxiliary requirement that 〈*ψ*|*L*_*t*_|*ψ*〉 = 0, whenever *ρ*|*ψ*〉 = 0. As already mentioned, as far as the definition of the SLD is concerned, we will actually confine ourselves to full-rank density matrices. In the case of singular density matrices, quantities of interest to us can be calculated *consistently* by a limiting procedure from the set of full rank matrices. In terms of *L*_*t*_, the “velocity” can be cast as $v=\sqrt{{\rm{T}}{\rm{r}}(\dot{w}{\dot{w}}^{\dagger })}=1/2\sqrt{{\rm{T}}{\rm{r}}[{L}_{t}\rho {L}_{t}]}$, which in turn means that the Bures metrics can be expressed in the following form

21 $${g}_{\mu \nu }=\frac{1}{8}{\rm{T}}{\rm{r}}(\rho \{{L}_{\mu },{L}_{\nu }\})$$

where *L*_*μ*_ is the restriction of *L*_*t*_ along the coordinate *λ*_*μ*_, and it is determined by the analog of equation (20), ${\partial }_{\mu }\rho =\frac{1}{2}\{{L}_{\mu },\rho \}$, where (${\partial }_{\mu }\,:\,=\partial /\partial {\lambda }_{\mu }$). We can also define the operator-valued differential one-form $L\,:\,={\sum }_{\mu }\,{L}_{\mu }d{\lambda }_{\mu }$. In the closed path *ρ*_*λ*(*t*)_, initial and final amplitudes are related by a unitary transformation, i.e. *w*_*λ*(*T*)_ = *w*_*λ*(0)_*V*_*γ*_. If the path of amplitudes *w*_*λ*(*t*)_ fullfills the Uhlmann condition, *V*_*γ*_ is a *holonomy*, the non-Abelian generalisation of Berry phase^13^. The holonomy is expressed as ${V}_{\gamma }={\mathscr{P}}{e}^{i{\oint }_{\gamma }A}$, where ${\mathscr{P}}$ is the path ordering operator and *A* is the Uhlmann connection one-form. The Uhlmann connection can be derived from the following ansatz^56^

22 $$dw+iwA=\frac{1}{2}Lw$$

which is the generalisation of (19) when the parallel transport condition is lifted. By differentiating $\rho =w{w}^{\dagger }$ and using the defining property of the SLD (see eq. (20)), it follows that *A* is Hermitian and it is implicitly defined by the equation

$$A{w}^{\dagger }w+{w}^{\dagger }wA=i({w}^{\dagger }dw-d{w}^{\dagger }w),$$

with the auxiliary constraint that 〈*ψ*′|*A*|*ψ*′〉 = 0, for *w*|*ψ*′〉 = 0. From eq. (22), it can be checked that *A* obeys the expected transformation rule of non-Abelian gauge potentials, $A\to {U}_{t}^{\dagger }A{U}_{t}+i{U}_{t}^{\dagger }d{U}_{t}$ under *w*_*t*_ → *w*_*t*_*U*_*t*_, and that *L* is gauge invariant.

The analog of the Berry curvature, the Uhlmann curvature two-form, is defined as $$F\,:\,=dA-iA\wedge A\,=$$$$\frac{1}{2}\,{\sum }_{\mu \nu }\,{F}_{\mu \nu }d{\lambda }_{\mu }\wedge d{\lambda }_{\nu }$$. Its components *F*_*μν*_ = ∂_*μ*_*A*_*ν*_ − ∂_*ν*_*A*_*μ*_ − *i*[*A*_*μ*_, *A*_*ν*_] can be understood in terms of the Uhlmann holonomy per unit area associated to an infinitesimal loop in the parameter space. Indeed, for an infinitesimal parallelogram *γ*_*μν*_, spanned by two independent directions $${\hat{e}}_{\mu }{\delta }_{\mu }$$ and $${\hat{e}}_{\nu }{\delta }_{\nu }$$ in the manifold, it reads

$${F}_{\mu \nu }=\mathop{\mathrm{lim}}\limits_{\delta \to 0}\,i\frac{1-{V}_{{\gamma }_{\mu ,\nu }}}{{\delta }_{\mu }{\delta }_{\nu }},$$

where *δ* → 0 is a shorthand of (*δ*_*μ*_, *δ*_*ν*_) → (0, 0).

As already mentioned, the Uhlmann geometric phase is defined as

23 $${\phi }^{U}[\gamma ]\,:\,=\arg ({w}_{\lambda (0)},{w}_{\lambda (T)})=\arg \,{\rm{T}}{\rm{r}}({w}_{\lambda (0)}^{\dagger }{w}_{\lambda (T)}),$$

and the Uhlmann phase per unit area for an infinitesimal loop reads

$${{\mathscr{U}}}_{\mu \nu }\,:\,=\mathop{{\rm{l}}{\rm{i}}{\rm{m}}}\limits_{\delta \to 0}\frac{{\phi }^{U}[{\gamma }_{\mu \nu }]}{{\delta }_{\mu }{\delta }_{\nu }}={\rm{T}}{\rm{r}}({w}_{\lambda (0)}^{\dagger }{w}_{\lambda (0)}{F}_{\mu \nu }).$$

We called the latter *mean Uhlmann curvature* (MUC), on account of the expression ${{\mathscr{U}}}_{\mu \nu }={\rm{Tr}}(\rho {F}_{\mu \nu })=\langle {F}_{\mu \nu }\rangle$ that ${\mathscr{U}}$ takes in the special gauge ${w}_{0}=\sqrt{\rho (0)}$.

By taking the external derivative of the expression (22) and by using the property *d*^2^ = 0, it can be shown that^56^

24 $$wF=\frac{i}{4}(L\wedge L-2dL)\,w,$$

25 $$F{w}^{\dagger }=\frac{i}{4}{w}^{\dagger }\,(L\wedge L+2dL).$$

Multiplying the above expressions by *w*^†^ and *w*, respectively, and taking the trace yields

26 $${\mathscr{U}}={\rm{T}}{\rm{r}}(wF{w}^{\dagger })=\frac{i}{4}{\rm{T}}{\rm{r}}(\rho L\wedge L),$$

where ${\mathscr{U}}\,:\,=1/2\,{\sum }_{\mu \nu }\,{{\mathscr{U}}}_{\mu \nu }d{\lambda }_{\mu }\wedge d{\lambda }_{\nu }$ is a real-valued two-form, whose components are ${{\mathscr{U}}}_{\mu \nu }=\frac{i}{4}{\rm{T}}{\rm{r}}(\rho [{L}_{\mu },{L}_{\nu }])$. The expressions of (21) and (26) reveal the common mathematical structure of MUC and metric tensor, which can be merged into a Hermitian matrix

27 $${I}_{\mu \nu }\,:\,={\rm{T}}{\rm{r}}(\rho {L}_{\mu }{L}_{\nu }),$$

called the quantum Fisher tensor (QFT)^72^, such that *g*_*μν*_ = **Re**(*I*_*μν*_)/4 and ${{\mathscr{U}}}_{\mu \nu }=-\,{\bf{I}}{\bf{m}}({I}_{\mu \nu })/2$.

### Fermionic Gaussian states

We will specialize our considerations to the case of systems described by fermionic Gaussian states. The fermionic Gaussian states are defined as density matrices *ρ* that can be expressed as

28 $$\rho \,=\,:{e}^{-\frac{i}{4}{{\boldsymbol{\omega }}}^{T}{\rm{\Omega }}{\boldsymbol{\omega }}}/Z,\,Z\,:\,={\rm{T}}{\rm{r}}{e}^{-\frac{i}{4}{{\boldsymbol{\omega }}}^{T}{\rm{\Omega }}{\boldsymbol{\omega }}}.$$

Here Ω is a 2*n* × 2*n* real antisymmetric matrix, and ${\boldsymbol{\omega }}\,:\,={({\omega }_{1}\ldots {\omega }_{2n})}^{T}$ is a vector of 2*n* Majorana fermion operators, defined as: ${\omega }_{2k-1}\,:\,={c}_{k}+{c}_{k}^{\dagger }$, ${\omega }_{2k}\,:\,=i({c}_{k}-{c}_{k}^{\dagger })$, with *k* = 1 … *n*, where *c*_*k*_ and ${c}_{k}^{\dagger }$ are annihilation and creation operators of standard fermions, respectively. The anticommutation relations of the Majorana fermion operators read {*ω*_*j*_, *ω*_*k*_} = 2*δ*_*jk*_. The Gaussian state is completely specified by the two-point correlation matrix ${{\rm{\Gamma }}}_{jk}\,:\,=1/2{\rm{Tr}}(\rho [{\omega }_{j},{\omega }_{k}])$, which is an imaginary antisymmetric matrix. One can show that Γ and Ω can be simultaneously cast in a canonical form by an orthogonal matrix *Q*

$${\rm{\Gamma }}=Q\underset{k=1}{\overset{n}{\oplus }}(\begin{array}{cc}0 & i{\gamma }_{k}\\ -i{\gamma }_{k} & 0\end{array}){Q}^{T},\,{\rm{\Omega }}=Q\underset{k=1}{\overset{n}{\oplus }}(\begin{array}{cc}0 & {{\rm{\Omega }}}_{k}\\ -{{\rm{\Omega }}}_{k} & 0\end{array}){Q}^{T},$$

and their eigenvalues are related by *γ*_*j*_ = tanh(Ω_*j*_/2), which implies that |*γ*_*j*_| ≤ 1. Correspondingly, the density matrix can be factorised as

$$\rho =\prod _{k=1}^{n}\,\frac{{\bf{1}}-i|{\gamma }_{k}|\,{z}_{2k-1}{z}_{2k}}{2},$$

where ${\boldsymbol{z}}={({z}_{1},\ldots ,{z}_{2n})}^{T}:\,=Q{\boldsymbol{\omega }}$ are the Majorana fermions in the eigenmode representation. Notice that |*γ*_*k*_| = 1 corresponds to the fermionic mode ${\tilde{c}}_{k}=1/2({z}_{2k-1}+{z}_{2k})$ being in a pure state.

For a Gaussian fermionic state, all odd-order correlation functions are zero, and all even-order correlations, higher than two, can be obtained from Γ by Wick’s theorem^73^, i.e. $${\rm{Tr}}(\rho {\omega }_{{k}_{1}}{\omega }_{{k}_{2}}\ldots {\omega }_{{k}_{2p}})={\rm{Pf}}({{\rm{\Gamma }}}_{{k}_{1}{k}_{2}\ldots {k}_{2p}})$$, where $$1\le {k}_{1} < \cdots  < {k}_{2p}\le 2n$$ and $${{\rm{\Gamma }}}_{{k}_{1}{k}_{2}\ldots {k}_{2p}}$$ is the corresponding 2*p* × 2*p* submatrix of Γ. $${\rm{Pf}}{({{\rm{\Gamma }}}_{{k}_{1}{k}_{2}\ldots {k}_{2p}})}^{2}=$$$${\rm{\det }}\,{\rm{Pf}}({{\rm{\Gamma }}}_{{k}_{1}{k}_{2}\ldots {k}_{2p}})$$ is the Pfaffian. An especially useful case is the four-point correlation function

29 $${\rm{Tr}}(\rho {\omega }_{j}{\omega }_{k}{\omega }_{l}{\omega }_{m})={a}_{jk}{a}_{lm}-{a}_{jl}{a}_{km}+{a}_{jm}{a}_{kl},$$

where ${a}_{jk}\,:\,={{\rm{\Gamma }}}_{jk}+{\delta }_{jk}$. We would like to derive a convenient expression for the QFT for Gaussian fermionic states. In order to do this, we first derive the SLD in terms of correlation matrix Γ. Due to the quadratic dependence of (28) in ***ω***, and following the arguments of^74^, it can be shown that *L* is a quadratic polynomial in the Majorana fermions

30 $$L\,=\,:\frac{1}{2}{{\boldsymbol{\omega }}}^{T}\cdot K{\boldsymbol{\omega }}+{{\boldsymbol{\zeta }}}^{T}{\boldsymbol{\omega }}+\eta ,$$

where $K={\sum }_{\mu }\,{K}_{\mu }d{\lambda }_{\mu }$, with *K*_*μ*_ a 2*n* × 2*n* hermitian antisymmetric matrix, ***ζ*** = ***ζ***_*μ*_*dλ*_*μ*_, with ***ζ***_*μ*_ a 2*n* real vector, and *η* = *η*_*μ*_*dλ*_*μ*_ a real valued one-form. From the property that Tr(*ρω*_*k*_) = 0 for any 1 ≤ *k* ≤ 2*n*, it is straightforward to show that the linear term in (30) is identically zero

$$0={\rm{T}}{\rm{r}}({\omega }_{k}d\rho )=\frac{1}{2}{\rm{T}}{\rm{r}}({\omega }_{k}\{L,\rho \})=\frac{1}{2}{\rm{T}}{\rm{r}}(\rho \{{{\boldsymbol{\zeta }}}^{T}{\boldsymbol{\omega }},{\omega }_{k}\})={\zeta }^{k}.$$

where *ζ*^*k*^ is the *k*-th component of ***ζ***, and in the third equality we took into account that the odd order correlations vanish. The quantity *η* can be determined from the trace preserving condition Tr(*dρ*) = Tr(*ρL*) = 0

31 $$\eta =-\,\frac{1}{2}{\rm{Tr}}(\rho \,{{\boldsymbol{\omega }}}^{T}K\,{\boldsymbol{\omega }})=\frac{1}{2}{\rm{Tr}}(K\cdot {\rm{\Gamma }}).$$

In order to determine *K*, we take the differential of Γ_*jk*_ = 1/2Tr(*ρ*[*ω*_*j*_, *ω*_*k*_])

32 $$\begin{array}{rcl}d{{\rm{\Gamma }}}_{jk}=\frac{1}{2}{\rm{Tr}}(d\rho [{\omega }_{j},{\omega }_{k}]) & = & \frac{1}{4}{\rm{Tr}}(\{\rho ,L\}[{\omega }_{j},{\omega }_{k}])\\  & = & \frac{1}{8}{\rm{Tr}}(\{\rho ,{{\boldsymbol{\omega }}}^{T}K{\boldsymbol{\omega }}\}[{\omega }_{j},{\omega }_{k}])+\eta \frac{1}{2}{\rm{Tr}}(\rho [{\omega }_{j},{\omega }_{k}])\\  & = & \frac{1}{16}\,\sum _{lm}\,{K}^{lm}{\rm{Tr}}(\rho \{[{\omega }_{l},{\omega }_{m}][{\omega }_{j},{\omega }_{k}]\})+\eta {{\rm{\Gamma }}}_{jk}\\  & = & {({\rm{\Gamma }}K{\rm{\Gamma }}-K)}_{jk}+[\eta -\frac{1}{2}{\rm{Tr}}(K\cdot {\rm{\Gamma }})]{{\rm{\Gamma }}}_{jk},\end{array}$$

where the last equality is obtained with the help of eq. (29) and using the antisymmetry of Γ and *K*. Finally, according to eq. (31), the last term vanishes and we obtain the following (discrete time) Lyapunov equation

33 $$d{\rm{\Gamma }}={\rm{\Gamma }}K{\rm{\Gamma }}-K.$$

The above equation can be formally solved by

$$K=-\,{({\bf{1}}-{{\rm{Ad}}}_{{\rm{\Gamma }}})}^{-1}(d{\rm{\Gamma }}),$$

where ${{\rm{Ad}}}_{{\rm{\Gamma }}}(X)\,:={\rm{\Gamma }}X{{\rm{\Gamma }}}^{\dagger }$ is the adjoint action. In the eigenbasis of Γ it reads

34 $${(K)}_{jk}=-\frac{{(d{\rm{\Gamma }})}_{jk}}{1-{\gamma }_{j}{\gamma }_{k}}=-\frac{d{{\rm{\Omega }}}_{k}}{2}{\delta }_{jk}+\,\tanh (\frac{{{\rm{\Omega }}}_{j}-{{\rm{\Omega }}}_{k}}{2})\langle j|dk\rangle ,$$

where, in the second equality, we made use of the relation *γ*_*k*_ = tanh(Ω_*k*_/2), which yields the following diagonal ${(d{\rm{\Gamma }})}_{jj}=(1-{\gamma }_{j}^{2})d{{\rm{\Omega }}}_{j}$ and off-diagonal terms (*d*Γ)_*jk*_ = (*γ*_*k*_ − *γ*_*j*_)〈*j*|*dk*〉. This expression is well defined everywhere except for *γ*_*j*_ = *γ*_*k*_ = ±1, where the Gaussian state *ρ* becomes singular (i.e. it is not full rank). In this condition, the expression (34) for the SLDs may become singular. Nevertheless, the boundness of the function $|\tanh \,\frac{{{\rm{\Omega }}}_{j}-{{\rm{\Omega }}}_{k}}{2}|\le 1$ in (34) shows that such a singularity is relatively benign. Thanks to this, we can show that the condition *γ*_*j*_ = *γ*_*k*_ = ±1 produces, at most, removable singularities in the QFT (cf.^57^). This allows the QFT to be extended by continuity from the set of full-rank density matrices to the submanifolds with *γ*_*j*_ = *γ*_*k*_ = ±1.

Knowing the expression for the SLDs, we can calculate the QFT by plugging ${L}_{\mu }=\frac{1}{2}[{{\boldsymbol{\omega }}}^{T}{K}_{\mu }{\boldsymbol{\omega }}-{\rm{Tr}}({K}_{\mu }\cdot {\rm{\Gamma }})]$ into ${I}_{\mu \nu }\,:\,={\rm{Tr}}(\rho {L}_{\nu }{L}_{\mu })$. Making use of (29) and exploiting the antisymmetry of both Γ and *K* leads to

35 $$\begin{array}{rcl}{I}_{\mu \nu } & = & \frac{1}{2}{\rm{Tr}}[({\bf{1}}+{\rm{\Gamma }}){K}_{\mu }({\bf{1}}-{\rm{\Gamma }}){K}_{\nu }]\\  & = & \frac{1}{2}\,\sum _{jk}\,(1+{\gamma }_{j})\,(1-{\gamma }_{k}){K}_{jk}{K}_{kj}\\  & = & \frac{1}{2}\,\sum _{jk}\,\frac{(1+{\gamma }_{j})\,(1-{\gamma }_{k})}{{(1-{\gamma }_{j}{\gamma }_{k})}^{2}}{({\partial }_{\mu }{\rm{\Gamma }})}_{jk}{({\partial }_{\nu }{\rm{\Gamma }})}_{kj},\end{array}$$

where the last equality is obtained by plugging in eq. (34). Let’s have a closer look at the QFT in the limit of (*γ*_*j*_, *γ*_*k*_) → ±(1, 1). The boundness *K*_*jk*_, and the multiplicative factors (1 ± *γ*_*j*_) in (35) causes each term with |*γ*_*j*_| → 1 to vanish. This means that the QFT has a well defined value in the above limit, and we can safely extend by continuity the QTF to the sub-manifolds (*γ*_*j*_, *γ*_*k*_) = ±(1, 1). The explicit expression of *I*_*μν*_ produces the following results for the Bures metrics

36 $${g}_{\mu \nu }=\frac{1}{4}{\bf{R}}{\bf{e}}({I}_{\mu \nu })=\frac{1}{8}{\rm{Tr}}({K}_{\mu }{K}_{\nu }-{\rm{\Gamma }}{K}_{\mu }{\rm{\Gamma }}{K}_{\nu })=-\,\frac{1}{8}{\rm{Tr}}({\partial }_{\mu }{\rm{\Gamma }}{K}_{\nu })=\frac{1}{8}\,\sum _{jk}\,\frac{{({\partial }_{\mu }{\rm{\Gamma }})}_{jk}{({\partial }_{\nu }{\rm{\Gamma }})}_{kj}}{1-{\gamma }_{j}{\gamma }_{k}},$$

which was already derived by Banchi *et al*.^41^. For the MUC the explicit expression is

37 $${{\mathscr{U}}}_{\mu \nu }=-\,\frac{1}{2}{\bf{I}}{\bf{m}}({I}_{\mu \nu })=\frac{i}{4}{\rm{Tr}}({\rm{\Gamma }}[{K}_{\mu },{K}_{\nu }])=\frac{i}{4}\,\sum _{jk}\,\frac{{\gamma }_{k}-{\gamma }_{j}}{{(1-{\gamma }_{j}{\gamma }_{k})}^{2}}{({\partial }_{\mu }{\rm{\Gamma }})}_{jk}{({\partial }_{\nu }{\rm{\Gamma }})}_{kj},$$

which, in a parameter-independent way, reads

38 $${\mathscr{U}}=\frac{i}{4}{\rm{Tr}}({\rm{\Gamma }}K\wedge K).$$

### Sufficient condition for criticality in translationally invariant dissipative models

In this section we will show that a singular dependence of ${\mathscr{U}}$ on the parameters $\lambda \in  {\mathcal M}$ necessarily implies a criticality, strictly in the sense of a diverging correlation length.

Let’s now prove that, in translationally invariant models, a vanishing dissipative gap is a *necessary condition* for criticality.

#### Proposition 1.

*If there exists a pole* ${\bar{z}}_{0}(\lambda )$ *of* $\tilde{\gamma }(z)$, *smoothly dependent of system parameters* $\lambda \in  {\mathcal M}$, *such that* ${\mathrm{lim}}_{\lambda \to {\lambda }_{0}}\,|{\bar{z}}_{0}|=1$, *then*

$${\rm{\Delta }}\,:\,=2\,{{\rm{\min }}}_{|z|=1,j}\,{\bf{R}}{\bf{e}}{x}_{j}(z)=0\,for\,\lambda ={\lambda }_{0}.$$

#### *Proof*.

Under the vectorising isomorphism, $A={a}_{jk}|j\rangle \langle k|\to {\rm{vec}}(A):\,={a}_{jk}|j\rangle \otimes |k\rangle$, the continuous Lyapunov equation (7) can be written as

39 $$\hat{X}(z){\rm{vec}}(\tilde{\gamma }(z))={\rm{vec}}(y(z)),$$

where $\hat{X}(z)\,:\,=x(z)\otimes {\bf{1}}+{\bf{1}}\otimes x({z}^{-1})$. When ${\rm{Det}}\hat{X}(z)\ne 0$, the unique solution of the symbol function is found simply as

40 $${\rm{vec}}(\gamma (z))=\frac{{\rm{vec}}(\eta (z))}{d(z)},\,{\rm{where}}\,{\rm{vec}}(\eta ):\,={\rm{adj}}(\hat{X}){\rm{vec}}(y).$$

Here ${\rm{adj}}(\hat{X})$ stands for the adjugate matrix of $\hat{X}$ and $d(z)\,:\,={\rm{Det}}\hat{X}(z)$. The point in writing the solution in this form, is that by construction, *x*(*z*) and *y*(*z*) are polynomials in *z* and *z*^−1^ with coefficients smoothly dependent on system parameters. Since determinant and adjugate matrix are always polynomial functions of matrix coefficients, it results that also *η*(*z*) and *d*(*z*) will be two polynomials in *z* and *z*^−1^. Hence, $\tilde{\gamma }(z)$’s poles are to be found among the roots $\bar{z}$ of *d*(*z*) = 0. Thus, a *necessary* condition for criticality is that, for *λ* → *λ*_0_, a given root $\bar{z}$ approaches the unit circle *S*_1_. This clearly means that for *λ* = *λ*_0_, there must exists ${\bar{z}}_{0}$ such that $|{\bar{z}}_{0}|=1$ and $d(z)={\rm{Det}}\hat{X}({\bar{z}}_{0})=0$, which implies a vanishing dissipative gap ${\rm{\Delta }}\,:\,=2\,{{\rm{\min }}}_{|z|=1,j}\,{\bf{R}}{\bf{e}}{x}_{j}(z)$, where *x*_*j*_(*z*)’s are the eigenvalues of $\tilde{x}(z)$^64^.◻

We will next show that a singular behaviour of ${\mathscr{U}}$ with respect to the parameters is a sufficient condition for criticality. First of all, notice, from the equation (13), that *u*(*ϕ*) may depend on the dynamics only through $\mathop{\gamma }\limits^{ \sim }$. Hence any closure of the gap which does not affect the analytical properties of $\tilde{\gamma }$ cannot result in a singular behaviour of ${\mathscr{U}}$ (see also proposition 2 in the following). We will just need to show that a necessary condition for a singular behaviour of *u*(*ϕ*) is Δ = 0.

Indeed, let’s now show that the poles of *u*_*μν*_(*z*) with |*z*| = 1 are to be found only among the roots of *d*(*z*). Assuming *d*(*z*) ≠ 0, and plugging the unique solution (40) into equation (13) leads to

$${u}_{\mu \nu }(z)=\frac{N(z)}{D(z)}=\frac{i}{4}\frac{d(z){\rm{Tr}}\{\eta (z)[{\partial }_{\mu }\eta (z),{\partial }_{\nu }\eta (z)]\}}{{(d{(z)}^{2}-{\rm{Det}}\eta (z))}^{2}},$$

where the numerator *N*(*z*) and denominator *D*(*z*) are polynomials in *z* and *z*^−1^ with smooth dependence on *λ*’s. We will demonstrate the following:

(i) that all roots of *d*(*z*) such that |*z*| = 1 are also roots of *D*(*z*);
(ii) that any other roots of *D*(*z*), such that |*z*| = 1, are not poles of *u*_*μν*_(*z*).

For the statement *(i)*, it is just enough to prove the following lemma.

#### Lemma 1.

*If d*(*z*) = 0 *with* |*z*| = 1, *then η*(*z*) = 0.

#### *Proof*.

For |*z*| = 1, let’s write explicitly *z* = *e*^*iϕ*^. It is not hard to show that from its definition, the matrix $\tilde{x}(\varphi )$ enjoys the following property $\tilde{x}{(\varphi )}^{\dagger }=\tilde{x}{(-\varphi )}^{T}$. Correspondingly, the eigenvalues of $\hat{X}$ are ${x}_{j}+{x}_{k}^{\ast }$ with *j*, *k* = 1, 2, where *x*_*j*_ are the eigenvalues of $\tilde{x}(\varphi )$. Since **Re***x*_*j*_ ≥ 0, ${\rm{Det}}\hat{X}=0$ implies that there must exist an eigenvalue *x*_0_ of $\tilde{x}(\varphi )$ with vanishing real part, hence ${\rm{\Delta }}=2\,{{\rm{\min }}}_{j}\,{\bf{R}}{\bf{e}}\,{x}_{j}=2\,{\bf{R}}{\bf{e}}\,{x}_{0}=0$. If |0〉 is the eigenstate of $\tilde{x}(\varphi )$ with eigenvalue *x*_0_, then

41 $${x}_{0}+{x}_{0}^{\ast }=\langle 0|\tilde{x}(\varphi )+\tilde{x}{(-\varphi )}^{T}|0\rangle =4\langle 0|\tilde{m}(\varphi )+\tilde{m}{(-\varphi )}^{T}|0\rangle =\mathrm{0\ },$$

where in the second equality we used the definition of $\tilde{x}(\varphi )\,:\,=2[2i\tilde{h}(\varphi )+\tilde{m}(\varphi )+{\tilde{m}}^{T}(\,-\,\varphi )]$ and the antisymmetry $\tilde{h}(\varphi )=-\,\tilde{h}{(-\varphi )}^{T}$. From the non-negativity of the $\tilde{m}(\varphi )$ matrices, it follows that $\langle 0|\tilde{y}(\varphi )|0\rangle =-\,4\langle 0|\tilde{m}(\varphi )-\tilde{m}{(-\varphi )}^{T}|0\rangle =0$. In^66^ it is shown that when 2**Re***x*_0_ = 0, the geometric multiplicity of *x*_0_ is equal to its algebraic multiplicity, hence the 2 × 2 matrix $\tilde{x}(\varphi )$ is diagonalisable. Then, let |*j*〉 be the set of eigenstates with eigenvalues *x*_*j*_. In the eigenbasis $|j\rangle \otimes |k\rangle$, *j*, *k* = 0, 1 the adjugate matrix has the following diagonal form,

$${\rm{a}}{\rm{d}}{\rm{j}}(\hat{X})=2(\begin{array}{cccc}|{x}_{0}+{x}_{1}^{\ast }{|}^{2}\,{\bf{R}}{\bf{e}}\,({x}_{1}) & 0 & 0 & 0\\ 0 & 2({x}_{0}+{x}_{1}^{\ast })\,{\bf{R}}{\bf{e}}\,({x}_{1}{x}_{0}) & 0 & 0\\ 0 & 0 & 2({x}_{1}+{x}_{0}^{\ast })\,{\bf{R}}{\bf{e}}\,({x}_{1}{x}_{0}) & 0\\ 0 & 0 & 0 & |{x}_{1}+{x}_{0}^{\ast }{|}^{2}\,{\bf{R}}{\bf{e}}\,({x}_{0})\end{array})$$

and due to **Re***x*_0_ = 0, all elements, but $\langle 0,0|{\rm{adj}}(\hat{X})|0,0\rangle$, vanish. On the other hand, the element ${\rm{vec}}{(\tilde{y})}_{00}\,:\,=\langle 0|\tilde{y}|0\rangle =0$, implying ${\rm{vec}}(\eta )={\rm{adj}}(\hat{X}){\rm{vec}}(y)=0$. ◻

To prove statement *(ii)*, we just need the following proposition.

#### Proposition 2.

*If* ${\bar{z}}_{0}$ *is a root of D*(*z*) *with* $|{\bar{z}}_{0}|=1$, *and* $d({\bar{z}}_{0})\ne 0$, *then u*_*μν*_(*z*) *is analytic in z*_0_.

#### *Proof*.

Let ${\bar{z}}_{0}$ be a root of *D*(*z*) with $|{\bar{z}}_{0}|=1$, with the assumption that $d({\bar{z}}_{0})\ne 0$. Notice that whenever *d*(*z*) ≠ 0, $\tilde{\gamma }(z)$ in (40) is the unique solution of the Lyapunov equation (7). As such, it is analytic in *z* (and smoothly dependent on *λ*’s). Since

42 $$D(z)\,:\,={(d{(z)}^{2}-{\rm{Det}}\eta (z))}^{2}=d{(z)}^{4}{[1-{\rm{Det}}\tilde{\gamma }(z)]}^{2},$$

we obviously have ${\rm{Det}}\tilde{\gamma }({\bar{z}}_{0})=1$. Just observe that if *γ*(*z*) is an analytic, smoothly dependent on the system parameters $\lambda \in  {\mathcal M}$, *u*_*μν*_(*z*) may be singular in ${\bar{z}}_{0}$ only if ${\rm{Det}}\tilde{\gamma }({\bar{z}}_{0})=1$. Assume then ${\rm{Det}}\tilde{\gamma }({\bar{z}}_{0})=1$, then either $\gamma ({\bar{z}}_{0})=\pm \,\,{\bf{1}}$. Without loss of generality, we can write $\tilde{\gamma }(z)={\bf{1}}+T{(z-{\bar{z}}_{0})}^{2n}+{\mathscr{O}}{(z-{\bar{z}}_{0})}^{2n}$, $n\in {\mathbb{N}}$, where $T={T}^{\dagger }$ is the first non-vanishing term of the Taylor expansion of $\tilde{\gamma }(z)-{\bf{1}}$. The fact that this term must be of even order (2*n*) is due to the positive semi-definiteness of the ${\bf{1}}-\tilde{\gamma }(z)$ for *z* ∈ *S*_1_. By expressing the 2 × 2 matrix *T* in terms of Pauli matrices, $T={t}_{0}{\bf{1}}+{\boldsymbol{t}}\cdot {\boldsymbol{\sigma }}$, where ${\boldsymbol{\sigma }}\,:\,={({\sigma }_{x},{\sigma }_{y},{\sigma }_{z})}^{T}$, ${t}_{0}\in {\mathbb{R}}$ and ${\boldsymbol{t}}\in {{\mathbb{R}}}^{3}$, the positive semi-definiteness condition above reads: *t*_0_ < 0 and ||***t***|| ≤ |*t*_0_|. Plugging the Taylor expansion in (13) and retaining only the first non-vanishing terms, yields    ◻

$${u}_{\mu \nu }(z)=-\,\frac{1}{4}\frac{{\boldsymbol{t}}\cdot ({\partial }_{\mu }{\boldsymbol{t}}\wedge {\partial }_{\nu }{\boldsymbol{t}})}{{t}_{0}^{2}}{(z-{\bar{z}}_{0})}^{2n}+o{(z-{\bar{z}}_{0})}^{2n}.$$

We have thus proven that a non-analycity of *u*_*μν*_(*z*) in ${\bar{z}}_{0}\in {S}_{1}$ is necessarily due to a pole $\bar{z}$ of $\tilde{\gamma }(z)$ approaching ${\bar{z}}_{0}$, as *λ* → *λ*_0_, resulting in a diverging correlation length. Therefore, a singular behaviour of $\bar{{\mathscr{U}}}$ in the manifold ${\mathcal M}$ is a sufficient criterion for criticality.

### The Mean Uhlmann Curvature and the quantum Fisher information matrix

As mentioned earlier, an important interpretation of ${\mathscr{U}}$ comes in the context of quantum metrology. The inverse *J*^−1^ of the quantum Fisher Information matrix (FIM), ${J}_{\mu \nu }=\frac{1}{2}{\rm{Tr}}\rho \{{L}_{\mu },{L}_{\nu }\}$, sets the quantum Cramér-Rao bound (CRB)^58,59^, i.e. a bound on the estimation precision of the parameters $\lambda \in  {\mathcal M}$ labelling a quantum state, i.e.

43 $${\rm{Cov}}(\hat{\lambda })\ge {J}^{-1},$$

where ${\rm{Cov}}{(\hat{\lambda })}_{\mu \nu }=\langle ({\hat{\lambda }}_{\mu }-{\lambda }_{\mu })({\hat{\lambda }}_{\nu }-{\lambda }_{\nu })\rangle$ is the covariance matrix of a set of locally unbiased estimators $\hat{\lambda }$ of the *λ*’s. The expression (43) should be understood as a matrix inequality. In general, one writes

$${\rm{tr}}(G{\rm{Cov}}(\hat{\lambda }))\ge {\rm{tr}}(G{J}^{-1}),$$

where *G* is a given positive definite cost matrix, which allows the uncertainty cost of different parameters to be weighed unevenly. In the case of the estimation of a single parameter *λ*_*μ*_, the above inequality can always be saturated, with the optimal measurement protocol being the projective measurement in the eigenbasis of the symmetric logarithmic derivative *L*_*μ*_. However, in the multi-parameter scenario, the CRB cannot always be saturated. Intuitively, this is due to the incompatibility of the optimal measurements for different parameters. A sufficient condition for the saturation is indeed [*L*_*μ*_, *L*_*ν*_] = 0, which is however not a necessary condition. Within the comprehensive framework of quantum local asymptotic normality (QLAN)^60–62^, a necessary and sufficient condition for the saturation of the multi-parameter CRB is given by ${{\mathscr{U}}}_{\mu \nu }=0$ for all *μ* and *ν*^53^.

Here, we show explicitly that ${{\mathscr{U}}}_{\mu \nu }$ provides a figure of merit for the discrepancy between an attainable multi-parameter bound and the single parameter CRB quantified by *J*^−1^. We will confine ourself to the broad framework of QLAN, in which the *attainable* multi-parameter bound is given by the so called Holevo Cramer-Rao bound (HCRB)^58,59^. For a *N*-parameter model, the HCRB can be expressed as^60^

44 $${\rm{tr}}(G{\rm{Cov}}(\hat{\lambda }))\ge {C}_{H}(G),$$

where

45 $${C}_{H}(G)\,:\,=\mathop{min}\limits_{\{{X}_{\mu }\}}\{{\rm{t}}{\rm{r}}(G\,{\bf{R}}{\bf{e}}\,Z)+\parallel (G\,{\bf{I}}{\bf{m}}Z){\parallel }_{1}\}.$$

The *N* × *N* Hermitian matrix is defined as ${Z}_{\mu \nu }\,:\,={\rm{Tr}}(\rho {X}_{\mu }{X}_{\nu })$, where {*X*_*μ*_} is an array of *N* Hermitian operators on ${\mathcal H}$ satisfying the unbiasedness conditions Tr(*ρX*_*μ*_) = 0 $\forall \,\mu$ and ${\rm{Tr}}({X}_{\mu }{\partial }_{\nu }\rho )=\frac{1}{2}{\rm{Tr}}\rho \{{X}_{\mu },{L}_{\nu }\}={\delta }_{\mu \nu }$ $\forall \,\mu ,\nu$, and ||*B*||_1_ denotes the sum of all singular values of *B*. If one chooses for {*X*_*μ*_} the array of operators ${\tilde{X}}_{\mu }\,:\,={\sum }_{\nu }\,{[{J}^{-1}]}_{\mu \nu }{L}_{\nu }$, it yields

46 $$Z=\,\tilde{Z}\,:\,={J}^{-1}I{J}^{-1}={J}^{-1}-i2{J}^{-1}{\mathscr{U}}{J}^{-1},$$

where ${I}_{\mu \nu }\,:\,={\rm{Tr}}\rho {L}_{\mu }{L}_{\nu }$ is the quantum Fisher tensor, and ${\mathscr{U}}$, with a little abuse of formalism, is the matrix of elements ${{\mathscr{U}}}_{\mu \nu }=\frac{i}{4}{\rm{Tr}}\rho [{L}_{\mu },{L}_{\nu }]$. If one indicates by ${\mathscr{D}}(G)\,:\,={C}_{H}(G)-{\rm{t}}{\rm{r}}(G{J}^{-1})$ the discrepancy between the attainable multi-parameter HCRB and the CRB, then ${\mathscr{D}}(G)$ is bounded as follows

47 $$0\le {\mathscr{D}}(G)\le 2\parallel G\,{J}^{-1}{\mathscr{U}}{J}^{-1}{\parallel }_{1},$$

where the first inequality is saturated iff ${\mathscr{U}}=0$^53^.

For the special case of a two-parameter model, in the eigenbasis of *J*, with eigenvalues *j*_1_ and *j*_2_, it holds

48 $${J}^{-1}{\mathscr{U}}{J}^{-1}=(\begin{array}{cc}{j}_{1}^{-1} & 0\\ 0 & {j}_{2}^{-1}\end{array})(\begin{array}{cc}0 & {{\mathscr{U}}}_{12}\\ -{{\mathscr{U}}}_{12} & 0\end{array})(\begin{array}{cc}{j}_{1}^{-1} & 0\\ 0 & {j}_{2}^{-1}\end{array})=(\begin{array}{cc}0 & \frac{{{\mathscr{U}}}_{12}}{{\rm{D}}{\rm{e}}{\rm{t}}J}\\ -\frac{{{\mathscr{U}}}_{12}}{{\rm{D}}{\rm{e}}{\rm{t}}J} & 0\end{array}).$$

It follows that

49 $$2\parallel G\,{J}^{-1}{\mathscr{U}}{J}^{-1}{\parallel }_{1}=2\sqrt{{\rm{D}}{\rm{e}}{\rm{t}}G}\,\frac{\sqrt{{\rm{D}}{\rm{e}}{\rm{t}}\,2{\mathscr{U}}}}{{\rm{D}}{\rm{e}}{\rm{t}}J}.$$

Hence, in this case $\sqrt{{\rm{Det}}\,2{\mathscr{U}}}/{\rm{Det}}J$ provides a figure of merit which measures the *amount of incompatibility* between two independent parameters in a quantum two-parameter model.

For self-adjoint operators *B*_1_, …, *B*_*N*_, the Schrodinger-Robertson’s uncertainty principle is the inequality^75^

50 $${\rm{D}}{\rm{e}}{\rm{t}}{[\frac{1}{2}{\rm{T}}{\rm{r}}(\rho \{{B}_{\mu },{B}_{\nu }\})]}_{\mu ,\nu =1}^{N}\ge {\rm{D}}{\rm{e}}{\rm{t}}{[\,-\frac{i}{2}{\rm{T}}{\rm{r}}(\rho [{B}_{\mu },{B}_{\nu }])]}_{\mu ,\nu =1}^{N},$$

which applied to the SLD *L*_*μ*_’s, yields

51 $${\rm{Det}}J\ge {\rm{Det}}2{\mathscr{U}}.$$

For *N* = 2, when the inequality (51) is saturated, it implies that

52 $${\mathscr{D}}(G)\simeq 2\sqrt{{\rm{Det}}G{J}^{-1}},$$

which means that the discrepancy ${\mathscr{D}}(G)$ reaches the same order of magnitude of tr(*GJ*^−1^), i.e. the CRB itself. This limit marks the *condition of maximal incompatibility* for the two-parameter estimation problem, arising from the quantum nature of the underlying system.

Another interesting inequality relates the eigenvalues of *J* and ${\mathscr{U}}$. The QFT ${I}_{\mu \nu }={\rm{T}}{\rm{r}}(\rho {L}_{\mu }{L}_{\nu })={J}_{\mu \nu }-i2{{\mathscr{U}}}_{\mu \nu }$ is a positive (semi)-definite Hermitian matrix. Hence, by definition $J\ge i2\,{\mathscr{U}}$, in a matrix sense. It follows that

53 $${j}_{i}\ge 2i\,{u}_{i},$$

where *j*_*i*_ and *u*_*i*_ are the i-th eigenvalues of *J* and ${\mathscr{U}}$, respectively, ordered according to ${j}_{1}\le {j}_{2}\le \cdots \le {j}_{N}$ and ${u}_{1}\le {u}_{2}\le \cdots \le {u}_{N}$. In particular, for i = 1, one gets

54 $$\parallel J{\parallel }_{\infty }\ge 2\parallel i\,{\mathscr{U}}{\parallel }_{\infty }.$$
